# General Factor of Personality and Its Relationship with the Dark Triad and Social Intelligence in Slovenian Adolescents

**DOI:** 10.3390/bs12090310

**Published:** 2022-08-27

**Authors:** Vesna Jug

**Affiliations:** Faculty of Mathematics, Natural Sciences and Information Technologies, University of Primorska, Glagoljaška 8, SI-6000 Koper, Slovenia; vesna.jug@upr.si

**Keywords:** general factor of personality (GFP), the Dark Triad, social intelligence, adolescence

## Abstract

The general factor of personality (GFP) represents the shared variance between personality traits that yield social adjustment and acts as a core personality disposition. In the present study, the existence of GFP in a sample of 249 Slovenian adolescents aged 15–19 years was investigated, and the relationship between GFP, the Dark Triad, and social intelligence was researched. The study used three self-report questionnaires to measure the Big Five, the Dark Triad, and three dimensions of social intelligence. It was found that, in adolescents, GFP exists (although in a somewhat different composition than in previous studies) and is negatively correlated with Machiavellianism and psychopathy and positively correlated with social skills and social awareness. GFP acts as a significant positive predictor of all social intelligence dimensions, and Machiavellianism acts as a significant positive predictor of social information processing and social skills. It can be concluded that GFP and, to a certain extent, perhaps some manipulative tendencies positively predict how an individual functions in society. With these findings, the study contributes to the understanding of the (hierarchical) structure of personality and its association with behavior in social interactions, which is one of the most important developmental tasks in adolescence.

## 1. Introduction

The concept of personality represents a comprehensive pattern of relatively permanent mental, behavioral, and physical characteristics by which individuals differ from each other [1]. In recent decades, the theory of five dimensions, which, at least since Goldberg [2], have been referred to as “the Big Five”—extraversion, agreeableness, conscientiousness, neuroticism, and openness—has grown in popularity [3,4,5]. The Big Five is considered to be fairly internally fragmented [6]. It is widely recognized but has been questioned by some researchers, as there is strong evidence that the dimensions are not orthogonal [7] and that there is a significant positive correlation between them.

One of the first researchers who dealt with the higher dimensions of personality was Digman [8]. Through factor analyses, later research indicated the existence of two higher-order factors above the Big Five (i.e., the Big Two). Recently [9], however, more and more findings indicate the existence of a single general personality factor (GFP). Musek [10] found that factor analyses extract a very strong first factor, which can be interpreted as high versus low emotional stability, agreeableness, conscientiousness, extraversion, and openness. GFP has been identified in many studies since its beginnings as a researched construct [11,12,13,14,15] and indicates whether an individual behaves in a socially acceptable or deviant manner.

GFP represents one of the most researched and debated topics in personality psychology [16]. In most studies, the existence and content of GFP have been confirmed [17], but it is also reasonable to mention studies that critically warn that GFP is only a methodological artifact tied to different statistical procedures [18,19,20,21]. Biesanz and West [22] noted that correlations between factors can only be established in data obtained with the help of self-assessment, while data obtained with the help of several assessors usually indicate orthogonal relationships between factors. Furthermore, Schermer and MacDougal [23] found that GFP is highly correlated with social desirability, and Rushton and Erdle [24] found that GFP still emerged after statistically controlling for social desirability, which indicates that GFP exists in the personality structure. In the vast majority of studies, the new proposed structural hierarchy of personality, which contains GFP at its top [9,10,11,12,13,14,15], has been verified. The results confirm the role of GFP in personality structure and thus suggest a revision of current prevailing structural models; therefore, a new hierarchical structural model of personality should be formulated [16].

Research results have also shown that GFP is strongly positively associated with psychological well-being, self-esteem, and the fundamental dimensions of emotions and motivation [9]. Most research associates GFP with other socially desirable personality traits [10,17,25]. There has also been some research that examined and confirmed the existence of GFP in children and adolescents [26,27,28]. These studies, similar to studies on adult samples, confirm the positive associations of GFP with other pleasant characteristics and suggest that GFP is positively associated with higher levels of popularity and likability [26] and a more favorable character [27].

Much less research associates GFP with traits commonly perceived as socially malevolent. Within research on these “dark personalities” [29] and socially undesirable personality traits [30,31], three have attracted the most attention: psychopathy, Machiavellianism, and narcissism. The “Dark Triad” [29] has become an established term for them, and it emphasizes the connections between the three dark personality traits [29,30,32]. Psychopathy and narcissism derive from classic clinical definitions [33], but recently, various authors [34] have tried to translate them into subclinical variables that can also be studied in the general population [35]. Subclinical psychopathy is characterized by high impulsivity, callousness, interpersonal manipulation, exploitation, stimulus seeking, and low levels of empathy, anxiety, and remorse [29]. Machiavellianism is a construct that does not have clinical definitions. It is characterized by the manipulation and exploitation of others; cunning, cold emotions; and a lack of sincerity or ethical importance [36]. Subclinical or “normal” narcissism represents feelings of grandeur, entitlement, dominance, and superiority [37,38,39]. 

Petrides et al. [40] examined the relationships between the Dark Triad and trait emotional intelligence. They found significant negative correlations between Machiavellianism and psychopathy and emotional intelligence, and significant positive correlations between narcissism and emotional intelligence. Based on this, they suggested that perhaps narcissism does not belong to the malevolent cluster of personality traits. There are several studies that suggest an alternative Dark Triad structure, i.e., that Machiavellianism and psychopathy are one and the same construct [41,42] and that instruments measuring Machiavellianism are actually measuring psychopathy. Rauthmann and Kolar [43] noted that narcissism differs from the other two malevolent traits, as it also includes prosocial aspects in addition to negative ones. Recent studies also suggest that Machiavellianism and psychopathy share a core called the callous exploitation of others [44,45]. Short instruments that measure the Dark Triad are quite often used, but it turns out that either (1) they are actually two-dimensional—containing a core of Machiavellianism–psychopathy and narcissism [44]—or (2) it would be better if, instead of separate subscale scores, a total composite score were used [45]. This research thus supports the idea of a “Dark Dyad” and argues that Machiavellianism and psychopathy are the same construct and that narcissism is less malevolent than the Machiavellianism–psychopathy core. Kowalski et al. [46] found that Machiavellianism and psychopathy are significantly negatively correlated with GFP, while narcissism is not significantly correlated with GFP.

Recently, the concept of psychopathy has been extended to children and adolescents: antisocial adolescents with psychopathic traits have been found [47] to have more diverse and serious behavioral problems. Based on this, it could be concluded that, in order to determine the social appropriateness of behavior, which is also referred to by the GFP, it is reasonable to know the expression of unfavorable personality traits. However, it is also important to emphasize that, regardless of the subclinical nature of the Dark Triad, the average expression of these characteristics in the general population, even in adolescents, is not high—research reports average values ranging between 1/3 and 1/2 of all possible points [44,48], similar to what can be detected in the expression of other less favorable personality traits.

Social intelligence is an older concept, but one that has recently reemerged as a scientific research topic. The first to formulate the concept of social intelligence was Thorndike [49], who defined it as “the ability to understand and manage men and women, boys and girls—to act wisely in human relations” [49] (p. 228). The construct was later presented in a more scientific way by Thorndike’s son, Robert L. Thorndike [50]. Cantor and Kihlstrom [51] defined social intelligence as important knowledge for behaving in society and achieving social goals. Socially intelligent people make new friendships and other social ties more easily and have no problems with communication and adapting to social situations [52]. During adolescence, the individual’s relationships with peers, siblings, parents, and other adults change in quantity and quality, and the adolescent communicates more actively with the wider social environment [53]. For this reason, the acquisition of social knowledge, as encompassed by the concept of social intelligence, is of utmost importance for effective behavior in society.

Social intelligence is undoubtedly a multidimensional construct. Kosmitzki and John [54], for example, defined several components of social intelligence: sensitivity to the internal states of others, a general ability to deal with others, knowledge of the social world and social rules, insight and sensitivity in complex social situations, use of social techniques to manipulate others, perspective taking, and social adjustment. Given the multidimensional form of social intelligence, instruments have also been designed to measure several dimensions—e.g., cognitive, behavioral, etc. One of the frequently used scales in Europe is the Tromsø social intelligence scale [55]. The scale measures three areas of social intelligence: (a) social information processing, which refers to understanding and predicting the behavior and feelings of other people; (b) social skills, which represent the behavioral aspect of social intelligence and refer to entering into new social interactions and adapting to them; and (c) social awareness, which refers to how individuals are aware of events in social situations. To function competently in society, it is important to master all the mentioned dimensions of social intelligence. It is particularly important to emphasize the developmental aspect, namely, that individuals in adolescence and emerging adulthood are only just establishing themselves in the social world. Understanding the behavior of others, inclusion in social situations, and interpreting social situations in general are, therefore, very important for youth.

Previous research on youth has examined the links between personality and social development but not specifically in the area of social intelligence, which represents a more comprehensive view of behavior in the social world. For example, it was found that GFP could represent a measure of social efficiency. Loehlin [56] notes that empathy, sociability, and adjustment have the highest loadings on GFP. Van der Linden et al. [57] found that individuals with higher GFP scores perform better on social judgment tests. GFP is also associated with positive social outcomes such as likability and popularity [28,58]; individuals with higher GFP scores also have fewer conflicts with others in everyday social interactions. Similarly, it can be seen that several studies have examined the relationship between personality characteristics and emotional intelligence, which is, in certain aspects, related to social intelligence. Ghiabi and Besharat [59] and Antoñanzas [60] found that there is a significant positive relationship between extraversion, openness, agreeableness, conscientiousness, and emotional intelligence and a significant negative relationship between neuroticism and emotional intelligence. Extraversion and neuroticism may predict changes related to emotional intelligence in positive and negative directions, respectively. A meta-analysis of studies on the relationship between GFP and emotional intelligence [61] found that GFP is significantly correlated with emotional intelligence in adults; however, there are differences between adult and adolescent personality structures. Researchers [62] have found U-shaped age trends for agreeableness, conscientiousness, and openness and negative age trends for extraversion in participants between the ages of 3 and 20, meaning that the relationship between GFP and emotional intelligence in adolescents could be different than in adults. This led Kawamoto et al. [63] to conduct a study on adolescents, and they found that there is also a significant positive correlation between GFP and emotional intelligence in youth. Based on this, research that specifically examines the connection between personality and social intelligence in youth would be beneficial, as the findings would deepen the understanding of the broader field of development in adolescence.

Personality dimensions (and, consequently, GFP) and dark personality traits play an important role in adolescents’ relationships with different people and in different situations of interpersonal behavior. Thus, the purpose of this study was to examine personality characteristics and social intelligence in adolescents and thereby provide additional knowledge in the field of development and the (hierarchical) structure of personality and its association with behavior in social interactions, which is one of the most important developmental tasks in adolescence. The study aimed to investigate the existence of GFP in a sample of adolescents and determine its connection to the Dark Triad and social intelligence. Based on theory and previous research, the following hypotheses were set:

1. GFP can be identified in adolescents.
2. There is a negative correlation between GFP and the Dark Triad.
3. There is a positive correlation between GFP and social intelligence.
4. GFP and the Dark Triad are significant predictors of social intelligence.

## 2. Materials and Methods

### 2.1. Sample

In total, 249 adolescents (108 males, 141 females) between the ages of 15 and 19 participated in the study. The average age of the participants was 17.11 years, and the standard deviation was 1.28 years. The participants were secondary school and early university students of various programs in Slovenia.

### 2.2. Instruments

Three questionnaires were used:

- Big Five Inventory [64]. The development of the questionnaire was based on the classic qualifications of the five personality factors, extraversion, agreeableness, conscientiousness, neuroticism, and intellect/openness. The questionnaire consists of 44 items, combined into the indicated factors, and rated on a five-point scale (1–strongly disagree, 5–strongly agree). In all factors, a higher score indicates a higher expression of the trait. The questionnaire has clearly defined items and is suitable for individuals from middle adolescence onwards [65] and, according to research data [66], even for individuals from the age of 10. Cronbach’s α coefficients of reliability are as follows: extraversion, 0.86; agreeableness, 0.79; conscientiousness, 0.82; neuroticism, 0.87; intellect/openness, 0.83; and the overall reliability of the questionnaire is 0.83.
- Dirty Dozen [67]. The questionnaire consists of 12 items combined into the subclinical dimensions of Machiavellianism, psychopathy, and narcissism. The items are rated on a nine-point scale (1–strongly disagree, 9–strongly agree). In all dimensions, a higher score indicates a higher expression of the trait. The scale is very short and clear and has been shown in previous research [68] to be suitable for use in adolescents from the age of 12. Cronbach’s α reliability coefficients are as follows: Machiavellianism, 0.79; psychopathy, 0.77; narcissism, 0.84; and the overall reliability of the questionnaire is 0.86.
- Tromsø Social Intelligence Scale [55]. The scale consists of 21 items combined into the dimensions of social information processing, social skills, and social awareness. A seven-point scale (1–not typical for me at all, 7–very typical for me) is used. In all dimensions, a higher score indicates a higher expression of the trait. The scale is short and consists of clearly defined items. Research [69] has confirmed that it is also suitable for use on adolescents from 13 years of age. Cronbach’s α reliability coefficient is 0.81 for the social information processing dimension, 0.86 for the social skills dimension, and 0.79 for the social awareness dimension.

### 2.3. Procedure

The study was part of a wider study in the field of the personality characteristics of adolescents. The participants were recruited by sending an invitation to a wide sample of secondary schools and faculties throughout Slovenia. Students who responded to the invitation were included in the sample. Data collection was performed in a paper–pencil manner. The participants completed the questionnaires individually or in groups (in a classroom setting). In the beginning, all participants were assured that their data were anonymous and that there were no right or wrong answers in the questionnaires. In all testing sessions, the researcher was present, and they answered the participants’ questions and explained the dilemmas regarding the items in the questionnaires. The informed consent to participate in the research was submitted for review and signature. For minor participants, the consent was also read and signed by their parents/legal guardians. The participants were asked to answer honestly and to complete the questionnaires in their entirety. Prior to each questionnaire, instructions were provided. The completion of the questionnaires took approximately 15 min. 

### 2.4. Statistical Analysis

For the data analyses, the IBM SPSS Statistics package was used. For all statistical analyses, the level of significance was set at *p* < 0.050 (two-tailed). In the first step, the descriptive statistics were calculated. For the extraction of GFP, several exploratory factor analysis (EFA) and confirmatory factor analysis (CFA) procedures were performed, similar to Musek’s [10] sequence. In the first EFA procedure, all 44 BFI items were included (principal component analysis (PCA) method, Promax rotation). Next, a CFA procedure with all BFI items (PCA method, Promax rotation) for the single factor solution was performed, and the values for the extracted factor were saved in the database as a new variable (“GFP_CFA”). With the aim of obtaining loadings of the Big Five dimensions, an EFA procedure (PCA method, Promax rotation) with the Big Five dimensions was performed, and the values of the extracted first factor were saved into the database as a separate variable (“GFP_B5”). For calculating the correlations between the Big Five, between “GFP_CFA” and “GFP_B5”, and between various variables, Spearman’s ρ correlation coefficient was used. Finally, for calculating the predictive power of GFP and the Dark Triad for the level of social intelligence, linear regression analysis procedures were performed for all three criteria, i.e., the three dimensions of social intelligence.

## 3. Results

Table 1 presents descriptive statistics of the sample. It can be seen that the obtained means for the Big Five Inventory and the Tromsø Social Intelligence Scale are slightly higher, and for the Dirty Dozen questionnaire, they are slightly lower than the midpoint scores for the dimensions. The standard deviations are appropriate for all of the questionnaires. Most of the variables are not normally distributed, which indicates the use of nonparametric statistical procedures. 

Table 2 shows that the personality factors correlate significantly with each other. Significant positive correlations can be observed between the following pairs of variables: extraversion–agreeableness, extraversion–conscientiousness, and agreeableness–conscientiousness. Significant negative correlations can be observed between the following pairs: extraversion–neuroticism, agreeableness–neuroticism, and conscientiousness–neuroticism. The correlations between openness and the other four factors are not statistically significant. The obtained results suggest that a hierarchical structure superior to the Big Five might exist in the sample.

In the first EFA procedure for the extraction of GFP (with all BFI items), the KMO coefficient of sampling adequacy was 0.785, which places it in the “middling” range [70,71], and Bartlett’s test of sphericity was statistically significant (*p* < 0.001). According to the Kaiser–Guttman criterion, 12 components with eigenvalues above one should be retained. The most noticeable is the first component, which has a noticeably larger eigenvalue than the others; according to Cattell’s criterion, a single-factor solution would be suitable. The results for the 12 extracted components are presented in Table 3.

Results from the EFA procedure for the Big Five dimensions show that the KMO coefficient of sampling adequacy is 0.660, which places it in the “mediocre”, but still acceptable, range [70,71], and Bartlett’s test of sphericity is statistically significant (*p* < 0.001). Table 4 shows that, according to the Kaiser–Guttman criterion, two components with eigenvalues above one should be retained. Again, the first component has a noticeably larger eigenvalue than the others—twice as large as the second component.

**Figure 1:**
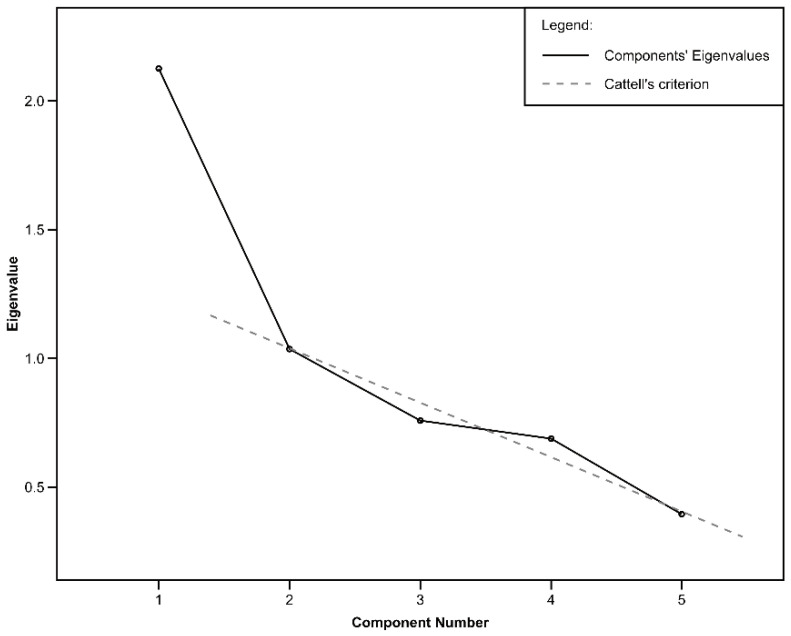
Scree plot for the five components of personality.

The scree plot in Figure 1 shows that the “jump” in the proportion of explained variance between the first and second component is obvious, a straight line could be drawn fairly well through the last four points. Cattell’s criterion, therefore, suggests one component. The first component explains more than forty percent of the total variance, which indicates the existence of a general factor. The loadings for the components are as follows: extraversion—0.675, agreeableness—0.762, conscientiousness—0.640, neuroticism—−0.821, and openness—0.065. The results, therefore, indicate a slightly different GFP structure, in which openness is not significantly represented.

“GFP_ CFA” and “GFP_B5” are very highly correlated (ρ = 0.967, *p* < 0.001), which is why, for greater clarity, the “GFP_B5” is used as a measure of the GFP in further analyses. “GFP_B5” contains all the Big Five dimensions, although the loading for openness is noticeably lower than loadings for other dimensions.

Table 5 shows that there are significant negative correlations between GFP and Machiavellianism, as well as psychopathy. In addition, GFP is significantly positively correlated with social skills and social awareness. There are also certain correlations between the Dark Triad and social intelligence—Machiavellianism is significantly positively correlated with social information processing, and psychopathy is significantly negatively correlated with social awareness.

The results of the regression analysis show that, for the “social information processing” criterion, R^2^ is 0.084 (effect size: Cohen’s f^2^ = 0.092); for the “social skills” criterion, R^2^ = 0.351 and f^2^ = 0.541; and for the “social awareness” criterion, R^2^ = 0.130 and f^2^ = 0.149. Table 6 shows that GFP is a significant predictor of all three dimensions of social intelligence. Machiavellianism appears as an important predictor of social information processing and social skills. A closer examination of the results shows that the β for the prediction of GFP for social information processing and for the prediction of Machiavellianism for social skills are quite low despite statistical significance. On the other hand, psychopathy and narcissism are not significant predictors of social intelligence.

## 4. Discussion

The main focus of the study was whether the existence of GFP in a sample of adolescents could be confirmed. The study also investigated the relationships between GFP, the Dark Triad, and social intelligence.

In the first hypothesis, the existence of GFP in adolescents was assumed. The results obtained through principal components analysis confirm the hypothesis to a certain extent—a strong first factor that explains more than forty percent of the variance was extracted, but the loadings show that openness is not significantly represented in it. The results of the study are partially consistent with previous research, which also confirmed the existence of GFP, both in adults [11,12,13,14,15] and in children and adolescents [26,27], but their GFP also contained a high loading of openness. In the present sample, four personality factors are included in the extracted GFP: extraversion, agreeableness, conscientiousness, and neuroticism (the latter in a negative direction). People who score high on the extracted GFP are sociable, self-confident, active, modest, productive, decisive, calm, positive, and have a high sense of order. The absence of a high loading of openness could partly be explained by a developmental point of view. To a certain extent, young people can be less open—they often join cliques and only accept their fellow members, as well as their ideas and values. They are less tolerant of nonmembers and do not always accept their differences [53]. This, however, does not explain the difference between the current and previous studies conducted on adolescents where GFP contained openness [26,27]. Perhaps these results could be explained with the help of a cross-cultural perspective. Previous research was performed on Dutch and US samples, and in these countries, compared with Slovenia, different values are important [72]. Even among the younger generations in Slovenia, materialist values are still somewhat more important compared to postmaterialist values. Factors such as economic growth, maintaining order, and the fight against crime are more important to people, while concepts such as freedom of speech, participation in government decisions, and the importance of ideas in society are less important. The importance of materialist versus postmaterialist values is also a sensitive indicator of whether survival or self-expression values are more important to people. Self-expression values are related to tolerance for different outgroups; people with such values assign a higher priority to tolerance for diversity, as well as environmental issues and participation in government decisions [72]. In the cross-cultural review of national surveys, it can be observed that Slovenia has slightly lower self-expression values scores compared to the Netherlands and the US. These cross-cultural differences, therefore, indicate that openness might be less pronounced in Slovenia, which could also help explain the results of the study—i.e., why openness does not correlate as strongly with other personality traits compared to samples from other countries.

In the second hypothesis, a negative correlation between GFP and the Dark Triad was assumed. The results confirm the hypothesis to a certain extent and show highly statistically significant negative correlations between GFP and Machiavellianism, as well as psychopathy, while the correlation between GFP and narcissism was not significant. The obtained results are completely consistent with the results of the study by Kowalski et al. [46]. Based on the results, it can be concluded that individuals who are more sociable, performance-oriented, decisive, order-loving, and positive-minded are less inclined to manipulate other people to achieve their own goals. Moral principles are also important to them, which they are reluctant to violate through their behavior. They are also characterized by a higher level of empathy and sensitivity to the well-being of others. At this point, it is also important to mention that the average values of the participants for dark personality traits are quite low, ranging between 1/3 and 1/2 of all possible points, which is also consistent with the findings of previous research [44,48]. This suggests that, for most of the study participants, their understanding of the social world and behavior in society can be considered to be relatively favorable.

The third hypothesis refers to the existence of a positive correlation between GFP and social intelligence. The results mostly confirm the hypothesis; strong positive correlations between GFP and social skills, as well as social awareness, are present. These results are consistent with the findings of previous research [28,57,58,61,63] that examined the correlations between GFP and various social outcomes and emotional intelligence. The obtained results can contribute to the understanding of this field, as they extend our knowledge about the relationships between GFP and other constructs into the field of social intelligence. Based on the results of the study, it can be concluded that individuals with better personal and social adjustment are self-confident in society, even if they do not know other people. They can adjust well to different social situations, easily establish new social contacts, and quickly find suitable topics for conversation. They easily understand other people’s decisions, and they know how to express their thoughts in a socially acceptable way. In social interactions, they behave in a way that does not hurt others. However, the obtained results did not confirm the correlation between GFP and social information processing. A closer examination of the data shows that there is a significant positive correlation between openness (which is not significantly represented in the extracted GFP) and social information processing. Based on this, it can be concluded that, compared with other personality aspects, openness is more important in understanding and predicting the emotions and behavior of other people.

In the fourth hypothesis, the GFP and Dark Triad were assumed to be important predictors of social intelligence. The results indicate that GFP is an important positive predictor of all three dimensions of social intelligence (social information processing, social skills, and social awareness). It can therefore be concluded that general personal and social adjustments play an important role in understanding the social world and socially acceptable behavior. A somewhat different picture regarding the predictive power of the Dark Triad can be seen: only Machiavellianism was found to be a significant predictor, while psychopathy and narcissism did not significantly predict social intelligence. Machiavellianism is thus an important positive predictor of social information processing and, to a somewhat lesser extent, also of social skills, but its predictive power is not significant to social awareness. These results are quite interesting, but they portray a slightly bleaker image of how society functions. They point to the fact that manipulative tendencies are an effective trait in the modern world—as if how we manipulate others and use them to our advantage is more important for understanding the social world than prosocial and altruistic traits. If we want to have a sustainable society and a just world, dark personality traits should not be desired. However, it is also important to re-emphasize that, in the study sample, a relatively small proportion of participants scored high on the Dark Triad, which is why it is necessary to be careful not to overestimate the positive connection between Machiavellianism and social information processing.

It is also important to look at the effect sizes of the regression models. The results show that the effect sizes are small when predicting social information processing and social awareness, but when predicting social skills, a large effect of the regression model can be observed. Based on this finding, it can be seen that GFP and, to a smaller extent, Machiavellianism are primarily important in developing the practical, behavioral aspect of social intelligence. An individual’s social skill level is primarily noticeable in their daily functioning in a social environment [55]. Based on the results of the study, it can be concluded that general personal and social adjustment, and perhaps some manipulative tendencies, are important for these concrete skills.

Finally, the results show the interesting fact that there is no significant correlation between GFP and social information processing, but GFP is an important predictor of this dimension of social intelligence. Something similar in the relationship between Machiavellianism and social skills could be observed. Based on the data, it is possible that this is a statistical issue and that GFP and Machiavellianism could act as suppressor variables. In the case of social information processing, adding GFP to the model raises the observed R^2^. GFP is significantly correlated with Machiavellianism, which is, in turn, significantly correlated with social information processing. GFP could therefore remove irrelevant predictive variance from Machiavellianism and increase its regression weight, resulting in a higher predictive value for the model [73]. Similar issues can be raised regarding Machiavellianism and its predictive value on social skills.

Based on the results of the study, it can be concluded that GFP exists in adolescents and is significantly associated with the Dark Triad and social intelligence. The conducted study possesses both theoretical and practical value. Considering that GFP represents a measure of social efficiency and is an important predictor of social skills as a dimension of social intelligence, it may be possible to predict how people will function in society based on their GFP level. Therefore, if lower GFP values were detected in youth, they could be included in various social skill training programs at an early age, which would help their functioning in society in the future. Making young people aware of the risks of the modern world—where certain socially undesirable traits can have great success—is of utmost importance. For the future of society, it is only acceptable that such behaviors not be used and that more prosocial traits be in the foreground. The improvement of social behavior would then also have a significant impact on friendships, the establishment of intimate relationships, and functioning in the professional environment.

The conducted study is not without limitations. A casual sample of participants was recruited for the study. These were students in secondary schools where management responded positively to the request to conduct the study and university students who were of the appropriate age and agreed to participate. Another limitation of the study refers to the fact that only self-assessment questionnaires were used, where various response biases could skew the results, and to a certain extent, the existence of GFP could even be overemphasized [22]. Some methodological limitations should also be mentioned. PCA, a widely used data dimensionality reduction analysis, was used for GFP extraction. The method expects a normal distribution of data, has some problems in analyzing outliers, and can sometimes maximize the variance of data. Perhaps it would be better to use latent class analysis (LCA), which includes discrete latent categorical variables that have a multinomial distribution and deals with the structure of groups. Another limitation is the fact that the results of several studies suggest the existence of a two-factor structure of dark personality traits, thus making the existence of the Dark Triad somewhat questionable. A limitation of the study is also that openness has a very low loading in the extracted GFP. Because of this, some relationships between variables and their interpretation could be slightly distorted and less accurate.

Further research in the field of personality structure, dark personality traits, and interpersonal intelligence in various developmental periods is of utmost importance. It may also be important to mention the possible redundancy between the Big Five and the Dark Triad: certain dark personality traits (or rather, perhaps, the absence of socially desirable traits) can also be found in the Big Five model, such as low-expressed agreeableness and conscientiousness. Similar characteristics can also be observed in Eysenck’s personality dimension of psychoticism [74]. Regardless of this, it is important to be aware of the existence of less socially desirable personality traits and to further measure them with instruments constructed specifically for them to determine their occurrence and structure in depth. Based on the findings of this study, it would be meaningful to further investigate the Dark Triad and, especially, the possibility of the existence of the Dark Dyad [40,41] or the existence of a single construct containing psychopathy and Machiavellianism [41,42,44,45]. It would also be significant to conduct additional research on the relationship between GFP and interpersonal (i.e., emotional and social) intelligence, as it is an area that is somewhat less researched but, therefore, no less important. It would also be meaningful to further explain the possible existence of mediator variables in the relationships between GFP, Machiavellianism, and social intelligence since there are undoubtedly variables that also contribute to the explanation of the development and level of social intelligence. Finally, broader, longitudinal research that explains how personality (including its dark characteristics), its (hierarchical) structure, and its association with other psychological constructs develop and change over the course of life would also be important.

## 5. Conclusions

The purpose of this study was to examine personality characteristics and social intelligence in adolescents. Summarizing the results, it can be seen that there are interesting relationships between GFP, the Dark Triad, and social intelligence. Many of the hypothesized relationships have been confirmed, and it can be concluded that GFP exists in adolescents but in a form that does not involve openness. GFP is negatively correlated with Machiavellianism and psychopathy and positively correlated with social skills and social awareness. GFP and Machiavellianism act as significant positive predictors of social information processing and social skills; GFP is also a significant positive predictor of social awareness. Therefore, it can be concluded that GFP and, to a certain extent, perhaps some manipulative tendencies positively predict how an individual functions in society.

## Figures and Tables

**Table 1:** Descriptive statistics.

	No. of Items	Min	Max	M	SD	Z ^1^	*p*
Extraversion	8	8	40	28.414	5.903	0.096	<0.001
Agreeableness	9	14	45	33.221	5.518	0.095	<0.001
Conscientiousness	9	10	45	29.060	6.589	0.066	0.010
Neuroticism	8	8	40	22.374	5.832	0.079	0.001
Openness	10	19	48	34.868	5.778	0.082	<0.001
Machiavellianism	4	4	36	12.366	6.831	0.128	<0.001
Psychopathy	4	4	31	11.582	6.433	0.127	<0.001
Narcissism	4	4	32	15.474	7.657	0.081	<0.001
Social information processing	7	14	48	32.855	6.535	0.071	0.004
Social skills	7	7	49	31.209	8.221	0.055	0.066
Social awareness	7	11	49	32.100	7.387	0.056	0.055

^1^ Kolmogorov–Smirnov coefficient.

**Table 2:** Correlation matrix of the Big Five.

	Extraversion	Agreeableness	Conscientiousness	Neuroticism
Extraversion	-			
Agreeableness	0.215 **	-		
Conscientiousness	0.297 **	0.316 **	-	
Neuroticism	−0.327 **	−0.494 **	−0.326 **	-
Openness	0.080	0.080	0.015	0.040

** *p* < 0.010.

**Table 3:** Explained variance in the 12 extracted components.

Component	Eigenvalue	% of Variance	Cumulative % of Variance
1	8.325	18.920	18.920
2	3.791	8.617	27.537
3	3.284	7.464	35.001
4	3.059	6.953	41.954
5	1.935	4.398	46.351
6	1.698	3.859	50.211
7	1.441	3.275	53.485
8	1.363	3.098	56.583
9	1.211	2.751	59.334
10	1.183	2.689	62.024
11	1.084	2.463	64.487
12	1.037	2.356	66.843

**Table 4:** Explained variance in the five components.

Component	Eigenvalue	% of Variance	Cumulative % of Variance
1	2.124	42.486	42.486
2	1.035	20.701	63.186
3	0.758	15.155	78.342
4	0.689	13.771	92.112
5	0.394	7.888	100.000

**Table 5:** Correlations between variables.

		1	2	3	4	5	6
1	General factor of personality (GFP)	-					
2	Machiavellianism	−0.281 **	-				
3	Psychopathy	−0.236 **	0.489 **	-			
4	Narcissism	−0.078	0.564 **	0.251 **	-		
5	Social information processing	0.121	0.258 **	0.087	0.114	-	
6	Social skills	0.524 **	−0.018	−0.098	−0.014	0.306 **	-
7	Social awareness	0.325 **	−0.110	−0.148 *	−0.031	−0.016	0.275 **

* *p* < 0.050. ** *p* < 0.010.

**Table 6:** Regression coefficients for individual predictors.

	Soc. Inf. Processing		Social Skills		Soc. Awareness	
Predictor	β	*p*	β	*p*	β	*p*
GFP	0.181	0.006	0.618	<0.001	0.335	<0.001
Machiavellianism	0.289	<0.001	0.197	0.004	0.071	0.365
Psychopathy	0.033	0.634	−0.031	0.586	−0.115	0.086
Narcissism	−0.030	0.675	−0.084	0.170	−0.020	0.782

β standardized regression coefficient.

## Data Availability

Not applicable.

## References

[1] Musek J. (2000). Nova Psihološka Teorija Vrednot.

[2] Goldberg L.R., Wheeler L. (1981). Language and individual differences: The search for universals in personality lexicons. Review of Personality and Social Psychology.

[3] Goldberg L.R. (1990). An alternative “description of personality”: The big five factor structure. J. Personal. Soc. Psychol..

[4] John O.P., Pervin L.A. (1990). The “big five” factor taxonomy: Dimensions of personality in the natural language and in questionnaires. Handbook of Personality: Theory and Research.

[5] Costa P.T., McCrae R.R. (1992). Four ways five factors are basic. Personal. Individ. Differ..

[6] Costa P.T., McCrae R.R., Dye D.A. (1991). Facet scales for agreeableness and conscientiousness: A revision of the NEO personality inventory. Personal. Individ. Differ..

[7] Becker P. (1999). Beyond the big five. Personal. Individ. Differ..

[8] Digman J.M. (1997). Higher-order factors of the big five. J. Personal. Soc. Psychol..

[9] Musek J. (2010). Psihologija Življenja.

[10] Musek J. (2007). A general factor of personality: Evidence for the big one in the five-factor model. J. Res. Personal..

[11] Rushton J.P., Irwing P. (2009). A general factor of personality (GFP) from the Multidimensional personality questionnaire. Personal. Individ. Differ..

[12] Rushton J.P., Irwing P. (2009). A general factor of personality in 16 sets of the big five, the Guilford–Zimmerman temperament survey, the California psychological inventory, and the Temperament and character inventory. Personal. Individ. Differ..

[13] Rushton J.P., Irwing P. (2009). A general factor of personality in the Millon clinical multiaxial inventory-III, the Dimensional assessment of personality pathology, and the Personality assessment inventory. J. Res. Personal..

[14] Erdle S., Rushton J.P. (2010). A general factor of personality, BIS-BAS, expectancies of reward and punishment, self-esteem, and positive and negative affect. Personal. Individ. Differ..

[15] Veselka L., Just C., Jang K.L., Johnson A.M., Vernon P.A. (2012). The general factor of personality: A critical test. Personal. Individ. Differ..

[16] Musek J. (2017). The general factor of personality: Ten years after. Psihol. Teme.

[17] Van der Linden D., Te Nijenhuis J., Bakker A.B. (2010). The general factor of personality: A meta-analysis of big five intercorrelations and a criterion-related validity study. J. Res. Personal..

[18] Schermer J.A., Holden R.R. (2019). Personality facet loadings onto the general factor of personality change when social desirability responding is considered. Personal. Individ. Differ..

[19] Chang L., Connelly B.S., Geeza A.A. (2012). Separating method factors and higher order traits of the big five: A meta-analytic multitrait-multimethod approach. J. Personal. Soc. Psychol..

[20] Ashton M.C., Lee K., Goldberg L.R., de Vries R.E. (2009). Higher order factors of personality: Do they exist?. Personal. Soc. Psychol. Rev..

[21] Revelle W., Wilt J. (2013). The general factor of personality: A general critique. J. Res. Personal..

[22] Biesanz J.C., West S.G. (2004). Towards understanding assessments of the big five: Multitrait–multimethod analyses of convergent and discriminant validity across measurement occasion and type of observer. J. Personal..

[23] Schermer J.A., Macdougall R. (2012). A general factor of personality, social desirability, cognitive ability, and the survey of work styles in an employment selection setting. Personal. Individ. Differ..

[24] Rushton J.P., Erdle S. (2009). No evidence that the social desirability response set explains the general factor of personality and its affective correlates. Twin Res. Hum. Genet..

[25] Erdle S., Irwing P., Rushton J.P., Park J. (2010). The general factor of personality and its relation to self-esteem in 628,640 internet respondents. Personal. Individ. Differ..

[26] Van der Linden D., Vreeke L., Muris P. (2013). Don’t be afraid of the general factor of personality (GFP): Its relationship with behavioral inhibition and anxiety symptoms in children. Personal. Individ. Differ..

[27] Dunkel C.S., Van der Linden D. (2017). The general factor of personality and character: A reanalysis. J. Genet. Psychol..

[28] Van der Linden D., Scholte R.H.J., Cillessen A.N.H., Te Nijenhuis J., Segers E. (2010). Classroom ratings of likeability and popularity are related to the big five and the general factor of personality. J. Res. Personal..

[29] Paulhus D.L., Williams K. (2002). The dark triad of personality: Narcissism, Machiavellianism, and psychopathy. J. Res. Personal..

[30] Lee K., Ashton M.C. (2005). Psychopathy, Machiavellianism, and narcissism in the five-factor model and the HEXACO model of personality structure. Personal. Individ. Differ..

[31] Kowalski R.M. (2001). Behaving Badly: Aversive Behaviors in Interpersonal Relationships.

[32] Vernon P.A., Villani V.C., Vickers L.C., Harris J.A. (2008). A behavioral genetic investigation of the dark triad and the big 5. Personal. Individ. Differ..

[33] Cleckley H. (1976). The Mask of Sanity.

[34] Levenson M.R. (1992). Rethinking psychopathy. Theory Psychol..

[35] Hare R.D. (1991). The Hare Psychopathy Checklist—Revised.

[36] Christie R., Geis F.L. (1970). Studies in Machiavellianism.

[37] Raskin R.N., Terry H. (1988). A principal-components analysis of the narcissistic personality inventory and further evidence of its construct validity. J. Personal. Soc. Psychol..

[38] Raskin R.N., Hall C.S. (1979). A narcissistic personality inventory. Psychol. Rep..

[39] Morf C.C., Rhodewalt F. (2001). Expanding the dynamic self-regulatory processing model of narcissism: Research directions for the future. Psychol. Inq..

[40] Petrides K.V., Vernon P.A., Schermer J.A., Veselka L. (2011). Trait emotional intelligence and the dark triad traits of personality. Twin Res. Hum. Genet..

[41] McHoskey J.W., Worzel W., Szyarto C. (1998). Machiavellianism and psychopathy. J. Personal. Soc. Psychol..

[42] Miller J.D., Hyatt C.S., Maples-Keller J.L., Carter N.T., Lynam D.R. (2017). Psychopathy and Machiavellianism: A distinction without a difference?. J. Personal..

[43] Rauthmann J.F., Kolar G.P. (2012). How “dark” are the dark triad traits? Examining the perceived darkness of narcissism, Machiavellianism, and psychopathy. Personal. Individ. Differ..

[44] Kajonius P.J., Persson B.N., Rosenberg P., Garcia D. (2016). The (mis)measurement of the Dark triad dirty dozen: Exploitation at the core of the scale. PeerJ.

[45] Persson B.N., Kajonius P.J., Garcia D. (2016). Revisiting the structure of the short dark triad. Assessment.

[46] Kowalski C.M., Vernon P.A., Schermer J.A. (2016). The general factor of personality: The relationship between the big one and the dark triad. Personal. Individ. Differ..

[47] Salekin R.T., Frick P.J. (2005). Psychopathy in children and adolescents: The need for a developmental perspective. J. Abnorm. Child Psychol..

[48] Czarna A.Z., Jonason P.K., Dufner M., Kossowska M. (2016). The Dirty dozen scale: Validation of a Polish version and extension of the nomological net. Front. Psychol..

[49] Thorndike E.L. (1920). Intelligence and its uses. Harper’s Mag..

[50] Thorndike R.L. (1936). Factor analysis of social and abstract intelligence. J. Educ. Psychol..

[51] Cantor N., Kihlstrom J.F. (1987). Personality and Social Intelligence.

[52] Buzan T. (2002). The Power of Social Intelligence.

[53] Zupančič M., Svetina M., Umek L.M., Zupančič M. (2020). Socialni razvoj v mladostništvu in na prehodu v odraslost. Razvojna Psihologija: 3. Zvezek.

[54] Kosmitzki C., John O.P. (1993). The implicit use of explicit conceptions of social intelligence. Personal. Individ. Differ..

[55] Silvera D.H., Martinussen M., Dahl T.I. (2001). The Tromsø social intelligence scale, a self-report measure of social intelligence. Scand. J. Psychol..

[56] Loehlin J.C. (2012). The general factor of personality: What lies beyond?. Personal. Individ. Differ..

[57] Van der Linden D., Oostrom J.K., Born M.P., Van der Molen H.T., Serlie A.W. (2014). Knowing what to do in social situations: The general factor of personality and performance on situational judgment tests. J. Personal. Psychol..

[58] Pelt D.H.M., Van der Linden D., Dunkel C.S., Born M.P. (2020). The general factor of personality and daily social experiences: Evidence for the social effectiveness hypothesis. Personal. Individ. Differ..

[59] Ghiabi B., Besharat M.A. (2011). An investigation of the relationship between personality dimensions and emotional intelligence. Procedia Soc. Behav. Sci..

[60] Antoñanzas J.L. (2020). The Relationship of personality, emotional intelligence, and aggressiveness in students: A study using the Big five personality questionnaire for children and adults (BFQ-NA). Eur. J. Investig. Health Psychol. Educ..

[61] Van der Linden D., Pekaar K.A., Bakker A.B., Schermer J.A., Vernon P.A., Dunkel C.S., Petrides K.V. (2017). Overlap between the general factor of personality and emotional intelligence: A meta-analysis. Psychol. Bull..

[62] Soto C.J. (2016). The little six personality dimensions from early childhood to early adulthood: Mean-level age and gender differences in parents’ reports. J. Personal..

[63] Kawamoto T., Kubota A.K., Sakakibara R., Muto S., Tonegawa A., Komatsu S., Endo T. (2021). The general factor of personality (GFP), trait emotional intelligence, and problem behaviors in Japanese teens. Personal. Individ. Differ..

[64] John O.P., Srivastava S., Pervin L.A., John O.P. (1999). The big-five trait taxonomy: History, measurement, and theoretical perspectives. Handbook of Personality: Theory and Research.

[65] Zupančič M., Umek L.M., Zupančič M. (2020). Čustveni in osebnostni razvoj v mladostništvu ter na prehodu v odraslost. Razvojna Psihologija: 3. Zvezek.

[66] Soto C.J., John O.P., Gosling S.D., Potter J. (2008). The developmental psychometrics of big five self-reports: Acquiescence, factor structure, coherence, and differentiation from ages 10 to 20. J. Personal. Soc. Psychol..

[67] Jonason P.K., Webster G.D. (2010). The dirty dozen: A concise measure of the dark triad. Psychol. Assess..

[68] Pechorro P., Jonason P.K., Raposo V., Maroco J. (2021). Dirty dozen: A concise measure of dark triad traits among at-risk youths. Curr. Psychol..

[69] Gini G. (2006). Brief report: Adaptation of the Italian version of the Tromsø social intelligence scale to the adolescent population. J. Adolesc..

[70] Kaiser H.F., Rice J. (1974). Little Jiffy, Mark IV. Educ. Psychol. Meas..

[71] Dziuban C.D., Shirkey E.C. (1974). When is a correlation matrix appropriate for factor analysis? Some decision rules. Psychol. Bull..

[72] Inglehart R.F. (2018). Cultural Evolution: People’s Motivations are Changing, and Reshaping the World.

[73] Pandey S., Elliott W. (2010). Suppressor variables in social work research: Ways to identify in multiple regression models. J. Soc. Soc. Work. Res..

[74] Eysenck H.J., Eysenck S.B.G. (1975). Manual of the Eysenck Personality Questionnaire (Junior and Adult).

